# Functional Mapping of Adhesiveness on Live Cells Reveals How Guidance Phenotypes Can Emerge From Complex Spatiotemporal Integrin Regulation

**DOI:** 10.3389/fbioe.2021.625366

**Published:** 2021-04-07

**Authors:** Philippe Robert, Martine Biarnes-Pelicot, Nicolas Garcia-Seyda, Petra Hatoum, Dominique Touchard, Sophie Brustlein, Philippe Nicolas, Bernard Malissen, Marie-Pierre Valignat, Olivier Theodoly

**Affiliations:** ^1^LAI, Aix-Marseille University, CNRS, INSERM U1067 Adhésion Cellulaires et lnflammation, Turing Center for Living Systems, Marseille, France; ^2^Aix-Marseille University, CNRS, INSERM U1104 Centre d’immunologie de Marseille-Luminy, Marseille, France

**Keywords:** adhesiveness, integrin affinity, LFA-1, VLA-4, lymphocyte, crosstalk, cell migration, haptotaxis

## Abstract

Immune cells have the ubiquitous capability to migrate disregarding the adhesion properties of the environment, which requires a versatile adaptation of their adhesiveness mediated by integrins, a family of specialized adhesion proteins. Each subtype of integrins has several ligands and several affinity states controlled by internal and external stimuli. However, probing cell adhesion properties on live cells without perturbing cell motility is highly challenging, especially *in vivo*. Here, we developed a novel *in vitro* method using micron-size beads pulled by flow to functionally probe the local surface adhesiveness of live and motile cells. This method allowed a functional mapping of the adhesiveness mediated by VLA-4 and LFA-1 integrins on the trailing and leading edges of live human T lymphocytes. We show that cell polarization processes enhance integrin-mediated adhesiveness toward cell rear for VLA-4 and cell front for LFA-1. Furthermore, an inhibiting crosstalk of LFA-1 toward VLA-4 and an activating crosstalk of VLA-4 toward LFA-1 were found to modulate cell adhesiveness with a long-distance effect across the cell. These combined signaling processes directly support the bistable model that explains the emergence of the versatile guidance of lymphocyte under flow. Molecularly, Sharpin, an LFA-1 inhibitor in lymphocyte uropod, was found involved in the LFA-1 deadhesion of lymphocytes; however, both Sharpin and Myosin inhibition had a rather modest impact on adhesiveness. Quantitative 3D immunostaining identified high-affinity LFA-1 and VLA-4 densities at around 50 and 100 molecules/μm^2^ in basal adherent zones, respectively. Interestingly, a latent adhesiveness of dorsal zones was not grasped by immunostaining but assessed by direct functional assays with beads. The combination of live functional assays, molecular imaging, and genome editing is instrumental to characterizing the spatiotemporal regulation of integrin-mediated adhesiveness at molecular and cell scales, which opens a new perspective to decipher sophisticated phenotypes of motility and guidance.

## Significance Statement

The adaptation of immune cell migration to various microenvironmental conditions is arguably mediated by integrins, a family of specialized adhesion proteins, but only partially understood. The experimental mapping of integrin properties at the cell surface would be instrumental to unraveling the underlying adaptive mechanisms, but this task is difficult on live cells. Here, we developed a novel *in vitro* method using micron-size beads pulled by flow to functionally probe the local adhesiveness on the surface of live and motile cells, allowing an unprecedented adhesiveness mapping at the trailing and leading edges of human T lymphocytes. This non-invasive approach yields phenotypic information on VLA-4 and LFA-1 integrin adhesiveness at the cell scale, which is complementary to molecular immunostaining approaches assessing signaling processes but not their cell-scale phenotypic outcome. We observed that cell polarization processes enhance integrin-mediated adhesiveness toward cell rear for VLA-4 and cell front for LFA-1 and that bidirectional crosstalk between LFA-1 and VLA-4 modulates adhesiveness with long-distance action across the cell. These findings explain the emergence of complex phenotypes such as the bistable orientation of lymphocytes downstream or upstream under flow. We also challenged the role of candidate proteins Sharpin and Myosin in uropod detachment from ICAM-1. While Sharpin participated to this process, both Sharpin and Myosin had a limited impact on lymphocyte adhesiveness, suggesting that other proteins are involved in LFA-1 deactivation. Finally, an original quantitative 3D imaging allowed us to link molecular densities of high-affinity integrins with local adhesiveness. All in all, the combination of molecular and functional mapping allowed us to explain cell orientation under flow. We foresee that such complementary studies will be instrumental to explaining other migrating phenotypes, such as chemotaxis or haptotaxis.

## Introduction

Integrins form a large family of adhesion proteins that are widely expressed on immune cells and play crucial roles in the immune response. Much is known on how integrins mediate the adhesion and migration of immune cells within the blood and lymphatic systems, lymphoid organs, and inflamed tissues (Laudanna et al., 1996; Ley et al., 2007; Petri et al., 2008; Huttenlocher and Horwitz, 2011; Kolaczkowska and Kubes, 2013), and less on the role of integrins in guidance versus chemical and mechanical cues (Stevens and Jacobs, 2002; Bartholomäus et al., 2009; Valignat et al., 2013, 2014; Gorina et al., 2014; Dominguez et al., 2015; Missirlis et al., 2016; Anderson et al., 2018, 2019; Buffone et al., 2018; Hornung et al., 2020). Integrins are much more than adhesion molecules; they have several conformations of different affinities (Schürpf and Springer, 2011), and each conformational change is controlled by intracellular molecular signals (e.g., binding of Talins, Kindlins, and Sharpin) (Shattil et al., 2010; Alon and Shulman, 2011; Pouwels et al., 2013), external stimuli (e.g., encounter with ligands, force, and ionic interactions) (Kim et al., 2003; Alon and Dustin, 2007; Pasvolsky et al., 2008; Lefort et al., 2009; Hogg et al., 2011; Nordenfelt et al., 2016), and crosstalk signals between different integrin subtypes (Porter and Hogg, 1997; Chan et al., 2000; May et al., 2000; Uotila et al., 2014; Grönholm et al., 2016). Integrins can also sense external mechanical forces and trigger intracellular signaling pathways (Hogg et al., 2011), allowing them to control mechanotaxis via mechanosensing (Dixit et al., 2011; Artemenko et al., 2016; Hornung et al., 2020; Luo et al., 2020), the same way that G-protein receptors control chemotaxis via chemosensing. Hence, complex regulation networks of adhesion are at work in immune cells that express several subtypes of integrins, each with different ligands, affinities, avidities, or clustering properties. The quantitative spatiotemporal characterization of the density and affinity state of integrins on the whole-cell surface would be instrumental to shedding light on the link between integrin regulation and mechanisms of cell migration and guidance. However, characterizing integrin states on live cells without perturbing the phenotypes of interest is a difficult task. Furthermore, molecular information of integrin state does not yield the effective adhesiveness at the cell scale, which is directly relevant in linking adhesion to cell migration and guiding. Therefore, our goal here was to directly measure the adhesion properties of the surface of live crawling cells at a subcellular scale. We developed for this task a new method to probe local adhesion by pulling with hydrodynamic flow on micron-size beads coated with integrin ligands and attached to cells.

Lymphocyte recruitment from blood relies mainly on integrins LFA-1 (αLβ2) and VLA-4 (α4β1). VLA-4 integrins, together with selectins, mediate transient adhesion of cells circulating in the blood stream by mediating a rolling motion of cells on the walls of blood vessels (Alon et al., 1995). This slow rolling motion allows further bonding of LFA-1 integrins that are slower to engage to their ligand and yield stronger adhesion. LFA-1 and VLA-4 then participate to subsequent autonomous crawling of cells on vessel walls. These sequential functions of VLA-4 and LFA-1 are consistent with their affinities toward their respective ligands VCAM-1 and ICAM-1 expressed by endothelial cells (Salas et al., 2002; Chigaev and Sklar, 2012a). In contrast, the mechanisms underlying integrin control of chemotaxis (Heit et al., 2005), haptotaxis (King et al., 2016; Swaminathan et al., 2016; Luo et al., 2020), or rheotaxis (Valignat et al., 2013; Dominguez et al., 2015) are hardly explained. Integrins are manifestly playing a central role in leukocyte orientation versus flow, because LFA-1 and VLA-4 were shown to mediate opposite orientations versus flow for lymphocytes *in vitro* (Valignat et al., 2013, 2014; Dominguez et al., 2015; Buffone et al., 2018, 2019). The participation of mechanotransduction in the mechanism remains in turn under debate. Mechanotransduction was reported for neutrophil (Dixit et al., 2011; Niethammer, 2016) and for lymphocyte (Roy et al., 2018, 2020) orientation under flow, as well as for lymphocytes after flow arrest (Kim and Hammer, 2019), but the causal link between integrin mechanotransduction and rheotaxis has not been fully established yet. Alternatively, we proposed a molecular mechanism without mechanotransduction, in which integrins LFA-1 and VLA-4 mediate a bistable system (Hornung et al., 2020). The mechanism is based on several regulation processes of integrin affinity states that are operational independently of flow. The polarization of effector lymphocytes is reported to trigger high-affinity conformations of LFA-1 in cell front (Smith et al., 2005, 2007; Ghandour et al., 2007; Shulman et al., 2009; Hogg et al., 2011; Valignat et al., 2013, 2014; Hornung et al., 2020) and of VLA-4 in cell rear (Laudanna et al., 1996; Smith et al., 2007; Morin et al., 2008; Shulman et al., 2009; Pouwels et al., 2013; Hornung et al., 2020) on the one hand, and low affinity of LFA-1 in cell rear (Semmrich et al., 2005; Morin et al., 2008; Pouwels et al., 2013) and VLA4 in cell front (Rantal et al., 2011; Grönholm et al., 2016; Hornung et al., 2020) on the other hand. Integrin high-affinity states are further stabilized when integrins encounter a ligand-coated solid substrate (Schürpf and Springer, 2011; Ishibashi et al., 2015). Finally, crosstalks have been reported to be activating for VLA-4 toward LFA-1 (Chan et al., 2000; May et al., 2000) and inhibiting for LFA-1 toward VLA-4 (Porter and Hogg, 1997; Grönholm et al., 2016). Combinations of these processes allowed us to qualitatively explain how LFA-1 and VLA-4 can control cell orientation against the flow when the leading edge was better attached than the trailing edge, and vice versa. However, the functional efficiency of these processes in terms of effective spatiotemporal regulation of adhesion at the cell scale has not been directly assessed. A direct quantitative measurement of cell-surface adhesiveness at the cell scale during lymphocyte crawling is lacking to verify that a network controlling integrin affinity can modulate differential adhesiveness of cell edges and control cell-directed migration. Our new method to measure local adhesiveness is used here to shed light on the bistable model of versatile lymphocyte guidance under flow.

Various techniques have been developed to assess the state of integrins at the cell surface and to decipher signaling pathways and regulation mechanisms. Flow chamber experiments with substrates coated by integrin ligands have revealed the kinetic properties of individual bonds in the regime of low ligand density (Grabovsky et al., 2000), and the role of force in the formation of shear-resistant bonds in the regime of high ligand density (Simon and Goldsmith, 2002). This technique yielded quantitative functional information on integrin-mediated adhesion, but spatial information at the subcellular level was not accessible. Immunostaining imaging of integrin properties at the subcellular level revealed modulations of densities and affinities between cell frontal, central, or trailing zones, as well as between basal or dorsal sides for crawling leukocytes (Smith et al., 2005; Pouwels et al., 2013). Although instrumental in the field, these approaches are often limited to the study of fixed cells because many antibodies can perturb the phenotypes of adhesion and migration of live cells (Smith et al., 2005). An attempt to avoid this bias may consist in using small-molecule probes as reporters of integrin affinity or bending states. Fluorescently labeled small molecules have been used to measure real-time ligand–receptor interactions with integrins LFA-1 and VLA-4 (Chigaev et al., 2009, 2011; Chigaev and Sklar, 2012b) by conventional flow cytometry, but they were not used for live microscopy yet. Alternatively, transfection of cells allowed the engineering of cells with fluorescent integrins, and a particularly elegant system involved a FRET construct reporting integrin extension state (Morin et al., 2008). However, this FRET reporter was only applied to a cell line yet, and the FRET signal was relatively weak. Single-molecule tracking is another powerful technique to tackle the problem of integrin-binding affinity and attachment–detachment kinetics (Ishibashi et al., 2015), but it has not been implemented on primary cells either and has not been employed to study crawling cells or to the different poles of a polarized crawling cell.

Here, we developed an extended version of the flow chamber method, in which flow was used to test the attachments and detachments of micron-size beads coated with integrin ligands and bonded to different locations on the cells. This technique allowed a phenotypic mapping of the local adhesiveness controlled specifically by integrins LFA-1 and VLA-4 on live crawling primary lymphocytes. It revealed that signaling pathways of polarization and crosstalk induce strong adhesiveness along the cell axis and explain versatile guiding of lymphocytes under flow.

## Results

### Laminar Flow Assays to Test Global or Local Adhesion of Lymphocytes

**FIGURE 1:**
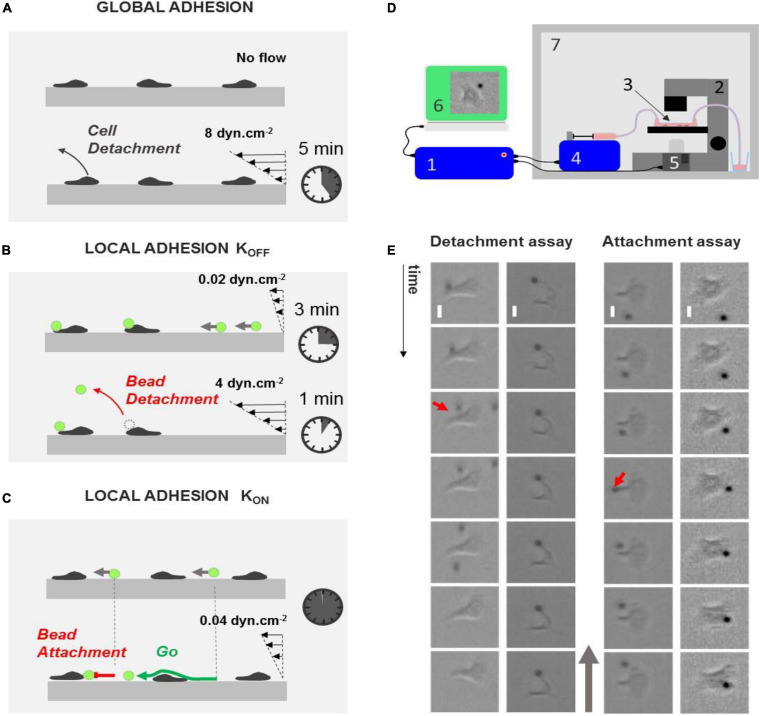
Laminar flow chamber to measure global and local adhesion. **(A)** Cartoon depicting the principle of flow chamber assay to probe cell adhesion. Cells (gray) are seeded without flow until they adhere and crawl. Their detachment under flow is then monitored versus time. **(B,C)** Cartoons depicting the principle of our flow chamber assays to probe local adhesion of the surface of cells using beads coated with CAM molecules (green). **(B)** Detachment experiment. Beads are injected at constant low flow, and they eventually adhered to cells upon random encounter. Flow is then increased, and bead detachments under flow are monitored versus time. **(C)** Attachment experiment. Beads are injected at a constant low flow, and their attachment frequency with cells is monitored. **(D)** Schematic of the automatized setup to repeat bead injection, multiple flow sequences, and image acquisitions. Schematics of the experimental setup. 1, controller; 2, optical microscope; 3, flow chamber; 4, controlled syringe pump; 5, controlled camera; 6, acquisition computer; 7, temperature-regulated cabinet. **(E)** Left—examples of events in detachment experiments with a bead remaining attached to lamellipods (left, from top to bottom: *t* = 0, 10, 20, 30, 40, 50, and 60 s) and a bead detaching from a cell uropod (right, from left to right: *t* = 0 s, 2500 ms, 5840 ms, 6000 ms, 12 s, and 24 s). Red arrow points to detachment. Scale bar 5 μm. Right—examples of events in attachment experiment with a bead attaching on lamellipods (left, from top to bottom: *t* = 0 s, 160 ms, 360 ms, 560 ms, 12 s, 24 s, and 36 s) and a bead passing along a cell uropod without attaching (right, from top to bottom: *t* = 0, 1, 2, 3, 4, 5, and 6 s). Red arrow points to attachment. Gray arrow indicates flow direction. Scale bar 5 μm.

Laminar flow chambers have been used to test cell adhesion *in vitro* on layers of endothelial cells or on substrates coated with cell adhesion molecules (CAMs). Shear stress of 1–10 dyn/cm^2^ is typically applied, and a qualitative estimation of global cell adhesion can be drawn from the fraction of cells that detach within a certain amount of time or from survival curves of adherent cells versus time (Figure 1A). These assays reveal functional defaults of global cell adhesion, which is relevant in diagnosing leukocyte adhesion deficiencies (LAD) (Robert et al., 2011). However, they provide no information at the subcellular level. To map the local adhesion strength of a cell surface, we developed here an extended version of laminar flow assays, which consisted in monitoring CAM-coated bead attachments on and detachments from live cells under flow (Figures 1B,C). Acquisitions were taken in 1 × 17 × 0.1-mm channels, and the flow sequences were controlled with an automatized syringe pump (Figure 1D). In detachment experiments, we repeatedly applied a sequence of flow at 0.02 dyn/cm^2^ for 3 min with a suspension of beads followed by a flow at 4 dyn/cm^2^ for 1 min with medium. The low shear sequence allowed the injection of a new batch of beads, whereas the high shear sequence was used to test the adherence of beads on cells. In attachment experiments, a single sequence of low shear at 0.04 dyn/cm^2^ was applied with a suspension of beads. Beads were rolling on the chamber bottom and randomly encountered crawling cells. The frequency of bead attachment was measured by the ratio of attachment events versus all encounter events.

We used here primary effector human T lymphocytes activated *in vitro* via CD3/CD28. Effector T lymphocytes crawled with a marked polarized shape consisting of a protruding lamellipod at cell leading edge and a contractile uropod at cell trailing edge. It was therefore easy to distinguish whether the events of bead attachment or detachment occurred at the trailing or leading edge of cells (Figure 1E and Supplementary Movie 1), which allowed probing adhesiveness differences along the front–rear polarization axis. The sensitivity and specificity of local force measurements were adjusted by modulating the nature and density of CAM on substrates and beads. ICAM-1 and VCAM-1, ligands of LFA-1 and VLA-4, respectively, were used at densities between 0 and 2400 molecules.μm^−2^. While encounters between cells and beads were random and equally probable on the trailing and leading edges of cells, attached beads were systematically advected toward cell trailing edge by rearward treadmilling of integrins linked to actin cytoskeleton (Aoun et al., 2020). Advection from cell leading to trailing edge lasted around 1 min. Consequently, pulling of beads was less frequent on cell leading edge than on cell trailing edge, and pulling on cell leading edge could only be monitored for less than a minute.

### Global Adhesion Is Stronger With LFA-1 Than With VLA-4

**FIGURE 2:**
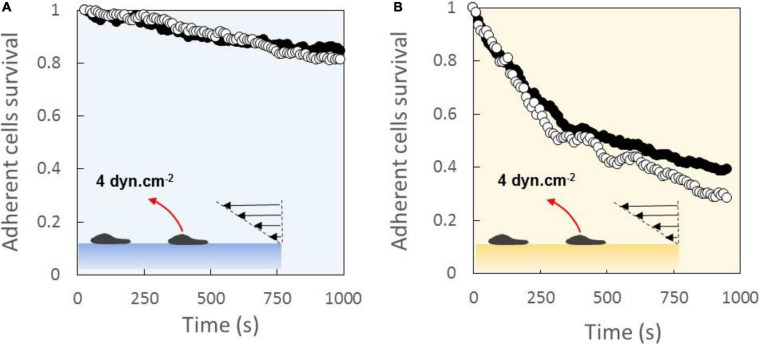
Laminar flow chamber experiments of global adhesion yield stronger binding with LFA-1 than with VLA-4. Survival versus time of cell bonds with a substrate coated with 1000 molecules.μm^−2^ (black dots) or 600 molecules.μm^−2^ (white dots) of ICAM-1 **(A)** and VCAM-1 **(B)**. Shear stress of 4 dyn/cm^2^ was continuous applied. Nexp = 3. N cells per experiment = 200.

Global adhesion of cells crawling on ICAM-1- (Figure 2A) and VCAM-1- (Figure 2B) coated substrates were probed by usual laminar flow experiments. Under a constant shear flow of 4 dyn/cm^2^, the survival curves of adherent cells revealed a stronger global adhesion on ICAM-1 than on VCAM-1. This effect is well established and consistent with *in vivo* observation of initial rolling mediated in part by VLA-4/VCAM-1 bonds and subsequent strong binding mediated by LFA-1/ICAM-1 bonds during the recruitment of leukocytes in blood vessels. Interestingly, the survival curves were identical for substrates coated with 1000 or 600 molecules.μ*m*^–2^ of CAM, although a higher probability of bond formation and a higher adhesion are possible. However, the average numbers of integrins measured by quantitative cytometry were 25,000 for LFA-1 and 15,000 for VCAM (Aoun et al., 2020), which corresponds to average densities of, respectively, 120 and 75 molecules.μ*m*^–2^ by considering an apparent cell diameter of 8 μm. Ligand densities on the substrates are therefore 5–15 times higher than integrin densities on cells, and the difference is even larger if one considers the real surface of cytoplasmic membrane (with submicronic microvilli) instead of the apparent surface assessed by optical microscopy. Hence, the independence of adhesion in this regime of CAM densities can be explained by an excess of ligands on substrates.

### LFA-1-Mediated Adhesion Is High in Cell Leading Edge and Low in Cell Trailing Edge

**FIGURE 3:**
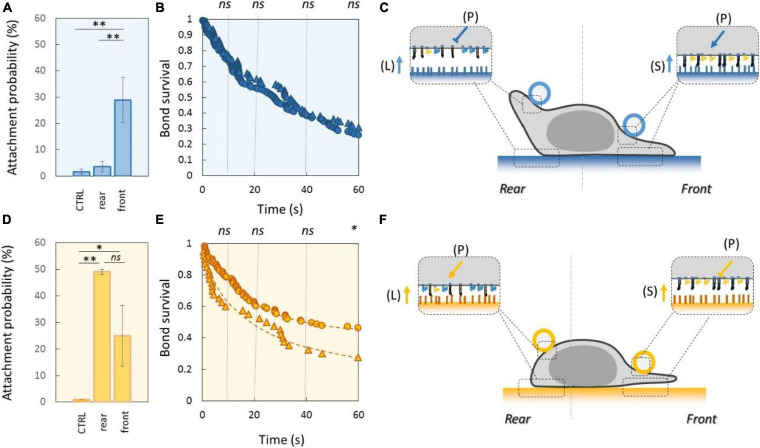
Polarization signaling triggers higher affinity of LFA-1 in leading edge and of VLA-4 in trailing edge. **(A–C)** Relate to beads and substrates coated with ICAM-1. **(D–F)** Relate to beads and substrates coated with VCAM-1. All substrates were coated with a CAM density of 1200 molecules.μm^−2^. **(A,D)** Attachment probability of beads with CAM densities of 2400 molecules.μm^−2^ on the rear and front of crawling cells. Controls (CTRL) correspond to data with control beads coated with IgG. Independent experiments Nexp = 4 in A and Nexp = 3 in D. Nevents_per_experiment > 40. **(B,E)** Survival curves of bead attached on cell rear (circles) or cell front (triangles). Blue dots refer to beads and substrates coated with ICAM-1, and orange dots to beads and surfaces coated with VCAM-1. Ligands density on beads was 2400 molecules.μm^−2^ for ICAM-1 and 480 molecules.μm^−2^ for VCAM-1. Nexp = 5, Nevents_per_exp > 90 (in B), Nexp = 4 (in E), Nevents_per_exp > 60 (in E). **(C,F)** Cartoons illustrating integrin control revealed in precedent data. Integrins are represented in orange for VLA-4 and blue for LFA-1, in their high-affinity and extended conformation (filled) and low-affinity bended conformation (hollow). Coatings on beads and substrates are colored in blue for ICAM-1 and orange for VCAM-1. Arrows indicate relative activating effect and T bars inhibiting effects for LFA-1 (orange) and VLA-4 (blue). Letter in bracket (P) indicates that signal originates from internal misopolarization. Error bars are standard deviations for independent experiments. ^∗^*P* < 0.05 and ^∗∗^*P* < 0.01, with respect to unpaired Student *t*-test. In B and E, statistical tests were performed at 10, 20, 40, and 60 s (dashed lines) and results indicated above the graphs.

Experiments with beads were then used to assess adhesiveness at the subcellular level. With beads and substrates coated with ICAM-1 (2400 molecules.μm^−2^ for beads and 1200 molecules.μm^−2^ for substrates), the attachment frequency was 32% on cell leading edge and 5% on cell trailing edge (Figure 3A). The lack of adhesiveness in cell rear cannot result from a lower density of integrins LFA-1 in cell rear because retrograde flow is constantly dragging integrins toward cell rear. Hence, although the survival curves of attached beads under flow revealed no significant difference between leading and trailing edges (Figure 3B), local adhesiveness results reveal that the cell polarization program is acting not only on the cell shape and on cytoskeleton dynamics but also on the spatial regulation of integrins affinity, here LFA-1 (Figure 3C). Furthermore, the inside-out signals from the polarization program, which are acting on integrins at molecular scale, are efficient enough to modulate adhesion properties at cell scale, but they do not have an all-or-nothing effect on integrins. Adhesiveness could be further increased by addition of Mn^2+^ at 3 mM (no detachment at 60 s). Interestingly, a lower LFA-1-mediated adhesiveness of cell rear is consistent with previous observations that uropods of effector lymphocytes are often detached on ICAM-1 substrates (Smith et al., 2005; Valignat et al., 2014; Hornung et al., 2020).

### VLA-4-Mediated Local Adhesion Is Lower in Cell Rear Than in Cell Front

With beads and substrates coated with VCAM-1 at 2400 and 1200 molecules.μm^−2^, respectively, the attachment frequency was 28% at the cell leading edge and 68% at cell trailing edge (Figure 3D). For detachment experiments, we decreased the density of VCAM-1 on beads fivefold to favor detachments, and survival curves also showed a slightly stronger adhesion in cell trailing edge than in cell leading edge (Figure 3E). These results suggest that the cell polarization signaling regulates the affinity of integrins, here VLA-4 (Figure 3F). This lower adhesion in cell front observed with VCAM-1 beads is consistent with previous observations of lamellipods loosely attached to the VCAM-1 substrate for effector lymphocytes (Hornung et al., 2020). Interestingly, polarization signaling has opposite effects on integrin upregulation in cell rear for VLA-4 and cell front for LFA-1. More surprisingly, local high adhesiveness was found stronger with VLA-4 on cell rear than for LFA-1 in cell front, whereas whole cells were globally more adherent on LFA-1 ligands than on VLA-4 (Figure 2). Like for LFA-1, polarization signaling did not have an all-or-nothing effect on integrins, because adhesiveness could be further increased by addition of Mn^2+^ 3 mM (no detachments at 60 s). Therefore, other stimuli can combine to polarization signaling to further modulate integrins state, like the presentation of integrin ligands by a substrate, which arguably favors integrin activation (King et al., 2016).

### Polarization Signals Control Integrin Affinity Independently of Cell Adhesion to Substrate

**FIGURE 4:**
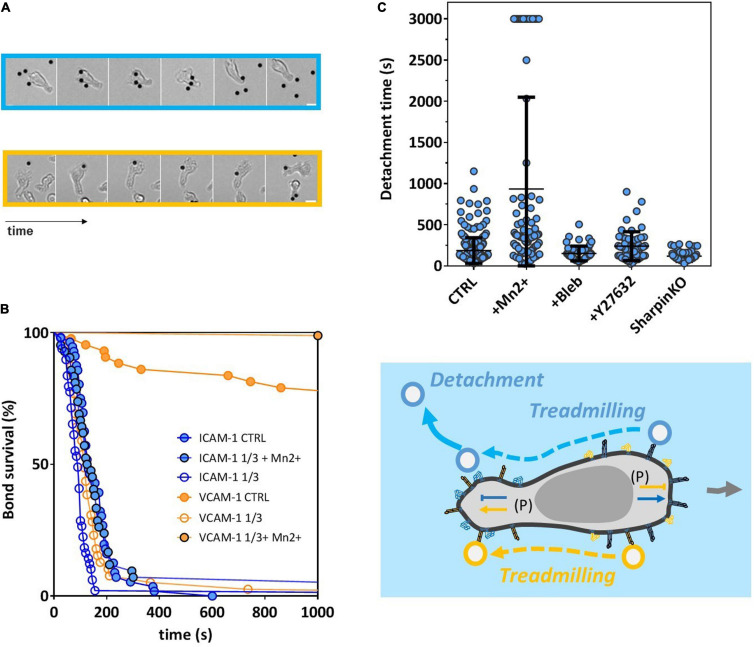
Polarization signals controlling integrin affinity are active independently of cell adhesion to a solid substrate. **(A)** Image sequence showing beads coated with ICAM-1 (top) or VCAM-1 (bottom) that attached to a cell front, treadmilled backward on cell body, and either detached spontaneously on cell rear for ICAM-1 beads or remained attached for VCAM-1 beads. From left to right, time is 15, 66, 78, 114, and 222 s for ICAM-1 and 300 s, and 0, 105, 135, 153, 177, and 333 s for VCAM-1. Scale bar is 5 μm (see also Supplementary Movie 2). **(B)** Bead/cell survival bonds for cells swimming without adhesion over a substrate passivated by Pluronics F127 and beads coated with either ICAM-1 (blue line) or VCAM-1 (orange line), with CAM densities of 2400 molecules/μm^−2^ for CTRL, 800 molecules/μm^−2^ for ICAM-1 and VCAM-1 1/3, and with or without Mn^2+^ at 3 mM (black contour dots). Number of independent experiment Ne = 3. Number of events per experiment > 50. **(C)** Bead/cell detachment times for wild cell beads coated with ICAM-1 at 2400 molecules/μm^−2^ in normal medium (CTRL) and with Mn^2+^ at 3 mM, blebbistatin at 50 mM, and Y27632 40 μM, and for Sharpin-deficient cells in normal medium. Independent experiment Nexp = 2. Events per experiment Ne > 50. **(D)** Cartoon of a swimming cell and beads coated with ICAM-1 (blue) or VCAM-1 (orange). Arrows outside cell indicate cell displacement. Arrows and T bars inside cell indicate, respectively, activating and inhibiting effects on LFA-1 (blue) and VLA-4 (orange). (P) indicates that signal comes from polarization processes.

It has been repeatedly observed that the presence of a solid substrate coated with integrin ligands was important or even required to trigger integrin high-affinity state (Schürpf and Springer, 2011; Nordenfelt et al., 2016) and allow subsequent cell adhesion, spreading, and crawling. To test whether spreading of cells on a solid substrate was required for the polarization machinery to selectively activate integrins, we performed experiments on cells suspended in solution without interaction with a solid substrate. These experiments were possible because effector T lymphocytes can maintain a polarized state in suspension and develop sustained directional motility by swimming (Aoun et al., 2020). Cells and beads were injected in a chamber treated with an antifouling Pluronic^®^ F-127 coating, in which they sedimented and swam in the vicinity of the substrate without adhesion. Encounters between the leading edge of swimming cells and immobile beads occurred randomly, and beads attaching cells were systematically dragged backward by the retrograde flow, until reaching the uropod (Figure 4A and Supplementary Movie 2). Survival curves were then established by counting the time lapse between the instant of first attachment of beads to cells front and the instant of detachment, if any (Figure 4B). Strikingly, ICAM-1-coated beads had a net tendency to detach spontaneously from cells’ uropod even though no force was exerted to pull them off in these experiments, with all beads detaching within 5 min. In contrast, VCAM-1 beads were much more strongly attached on cells’ uropod, 80% of VCAM-1 beads being still attached after 15 min. These results are consistent with a down- and upregulation of LFA-1 and VLA-4 in cell rear, respectively. We then found that attachments increased in the presence of Mn^2+^ 1 mM, which shows that polarization signals are not all or nothing and that they decreased with beads coated at CAM densities divided threefold, which confirms that our measurements are integrin specific. Then, treatments with blebbistatin or Y27632 showed hardly any effect on the detachment of ICAM-1 beads, which suggests that activation of Myosin II in cell rear (via ROCK/RhoA) has not a determinant role in LFA-1 de-adhesion of cell rear. This result is in line with some previous studies (Smith et al., 2003) and in opposition with others (Morin et al., 2008). In the latter studies, detachment defects of cell rear on ICAM-1 were inferred from the elongation of uropods, and such elongations may alternatively result from an increase of deformability induced by blebbistatin (Gabriele et al., 2009), without attachment alteration. Finally, we performed experiments on Sharpin-KO cells and found no strong effect on detachment, which is consistent with detachment experiment under flow. While molecular signaling remains unclear, these results show that the polarization-linked inside-out signals controlling integrin affinity are operational independently of the outside-in signaling induced by the spreading of a cell on a substrate bearing integrins ligands.

### SHARPIN Mediates Lower LFA-1 Adhesion and Modulates Local Adhesion in Both Leading and Trailing Edges of Effector Lymphocytes

**FIGURE 5:**
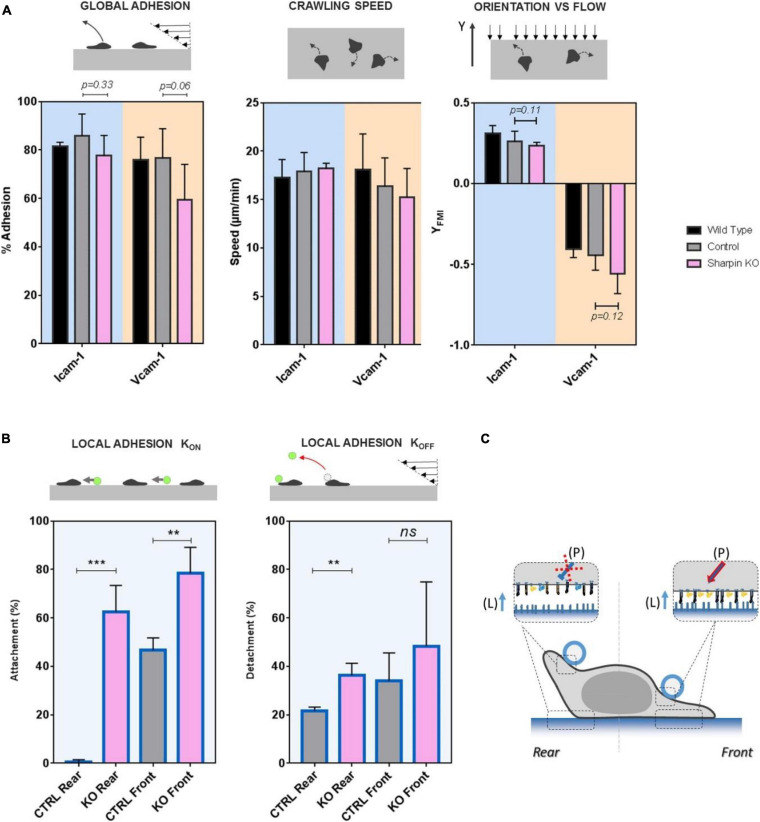
SHARPIN modulates effective adhesion in leading and trailing edges and with opposite effect on LFA-1 and VLA-4. **(A)** Global adhesion experiments. Left—percentage of cells adhered after 5 min of shear stress at 4 dyn/cm^2^. Center—speed of crawling cells. Right—forward migration index in the direction of flow (>0 against, >0 with) at 4 dyn/cm^2^. Substrates are coated with CAM-1 at 1200 molecules.μm^−2^. Background is blue for surface ICAM-1 and orange for VCAM-1. Color bar is black for wild-type cells, gray for control cells transfected with unspecific RNP complexes, and pink for Sharpin-deficient cells. Nexp = 3. Ncell > 200 per experiment. Error bars are standard deviations for independent experiments. *P*-values according to Student *t*-test. **(B)** Local adhesion experiments. Left—attachment probability and right—detachment after 40 s of flow of beads with ICAM-1 at 2400 molecules.μm^−2^ on the rear or front of cells crawling on substrates coated with ICAM-1 at 1200 molecules.μm^−2^. Gray for control cells transfected with unspecific RNP complexes and pink for Sharpin-deficient cells. Nexp = 3. Ncells > 30. Error bars are standard deviations for independent experiments. **P < 0.01 and ***P < 0.001, with respect to unpaired Student *t*-test. **(C)** Cartoon illustrating that perturbation in Sharpin-deficient cells. Integrins are represented in orange for VLA-4 and blue for LFA-1, in their high-affinity and extended conformation (filled) and low-affinity bended conformation (hollow). The red cross indicates that the inhibiting signal of LFA-1 is diminished in cell rear and the red arrow that the activating signal is enhanced in cell front. Letters in brackets indicate the origin of the signal to integrin either from internal polarization signaling (P) or ligand presented by a substrate (L). Error bars are standard deviations for independent experiments. **P < 0.01 and ***P < 0.001, with respect to unpaired Student *t*-test. Number of independent experiments Nexp = 3. Number of events per experiment Ne > 100 for attachment and > 40 for detachments.

Recent studies (Rantal et al., 2011; Pouwels et al., 2013) showed that protein Sharpin mediated an inhibiting signal of LFA-1 in the rear of crawling lymphocytes. To test the effect of Sharpin on adhesion at subcellular scale, we used CRISPR-Cas9 genome editing technology to generate Sharpin-deficient T lymphocytes (Supplementary Figure 1). These cells adhered on ICAM-1- and VCAM-1-coated substrates and crawled with unbiased speed as compared to control cells (Figure 5A). Global adhesion tested by flow experiments was also not significantly altered (Figure 5A). The orientation against flow, which is strongly dependent on an efficient detachment of uropod, was not significantly altered (Figure 5A), which suggests that Sharpin is not required for uropod detachment in effector T cells. Altogether, assays on global cell adhesion/migration/guidance revealed no critical effect of Sharpin. In assays of local adhesiveness with beads and flow, the attachment frequencies when both beads and substrates were coated with ICAM-1 were higher (Figure 5B) on both leading and trailing edges for Sharpin-deficient cells as compared to control-transfected cells, whereas detachments were slower on trailing edges (Figure 5B). These results are consistent with the hypothesis that Sharpin participates to LFA-1 deactivation in cell rear (Pouwels et al., 2013) and further suggest that this function also operates in cell front (Figure 5C). However, Sharpin deficiency seems to hardly hamper deadhesion. From a methodological point of view, it is interesting to note that slight perturbations of adhesiveness could be detected by local but not by global adhesion assays.

### Inhibiting Crosstalk of LFA-1 Toward VLA-4 and Activating Crosstalk of VLA-4 Toward LFA-1 Modulate Adhesion at Long Distances Across the Cell

**FIGURE 6:**
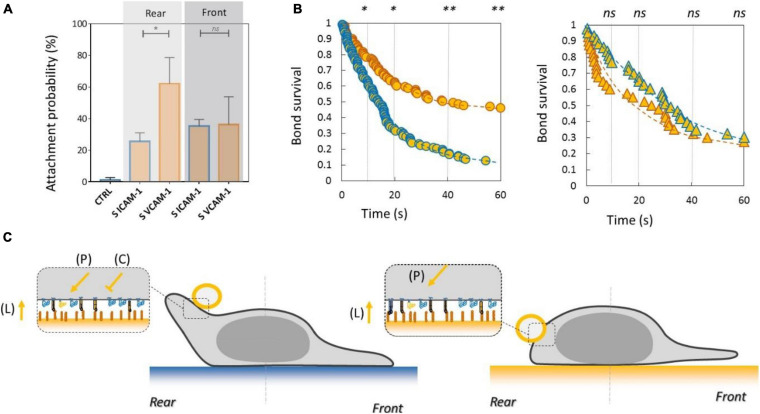
Inhibiting crosstalk of LFA-1 toward VLA-4 modulates cell surface adhesiveness at supramolecular scale across the cell. **(A)** Attachment probability of beads with VCAM-1 at 2400 molecules.μm^−2^ on the rear and front of cells crawling on substrates coated with ICAM-1 (S ICAM) or VCAM-1 (S VCAM) at 1200 molecules.μm^−2^. CTRL corresponds to beads coated with irrelevant IgG. Number of independent experiments Nexp = 3. Number of events_per_experiment Ne > 40. **(B)** Bead/cell survival bonds for beads coated by VCAM-1 at 480 molecules.μm^−2^ attached on cell rear (circles) or (on cell front (triangles) with cells crawling on substrates coated with ICAM-1 (marks circled in blue) and on VCAM-1 (marks circled in orange). Number of independent experiments Nexp = 4, number of events_per_experiment Ne > 70. Error bars are standard deviations for independent experiments. *P < 0.05 and **P < 0.01, with respect to unpaired Student *t*-test. In **B**, statistical tests were performed at 10, 20, 40, and 60 s (dashed lines) and results indicated above the graphs. **(C)** Cartoon of cell with bead and substrates coated with ICAM-1 (top) and VCAM-1 (bottom). Orange arrows and T-lines indicate activation and inhibiting effects on VLA-4, and letters indicate their origin, i.e., the polarization machinery (P), the presentation of ligand by a surface (L), and the crosstalk from LFA-1 (C).

**FIGURE 7:**
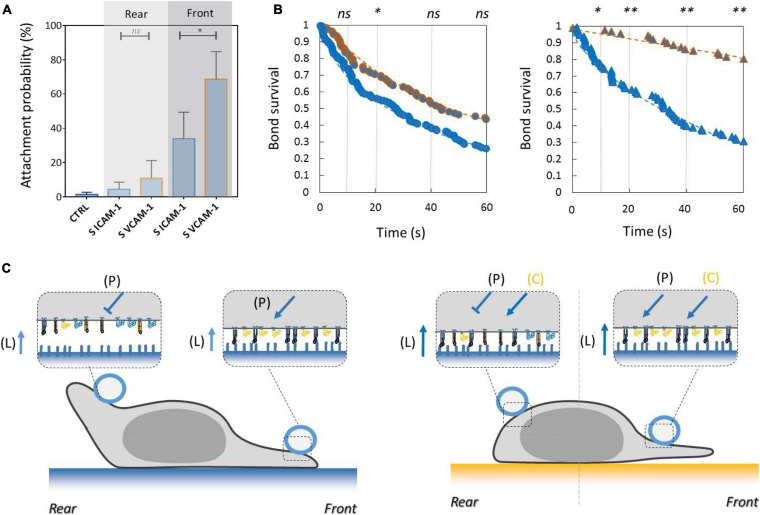
Activating crosstalk of VLA-4 toward LFA-1 modulates cell surface adhesiveness on long distances across the cell. **(A)** Attachment probability of beads with ICAM-1 at 2400 molecules.μm^−2^ on the rear and front of cells crawling on substrates coated with ICAM-1 (S ICAM) or VCAM-1 (S VCAM) at 1200 molecules.μm^−2^. CTRL corresponds to beads coated with irrelevant IgG. Number of independent experiments Nexp = 3. Number of events_per_experiment Ne > 40. **(B)** Survival versus time of bead/cell bonds formed on cell rear (circles) or on cell front (triangles) with cells crawling on substrates coated with ICAM-1 (marks circled in blue) and on VCAM-1 (marks circled in orange). Number of independent experiments Nexp = 4. Number of events_per_experiment Ne > 70. **(C)** Cartoon of cell with bead and substrates coated with ICAM-1 (top) and VCAM-1 (bottom). Orange arrows and T-lines indicate activation and inhibiting effects on VLA-4, and letters indicate their origin, i.e., the polarization machinery (P), ligand bonding (L), and crosstalk from LFA-1 (C). Error bars are standard deviations for independent experiments. ^∗^*P* < 0.05 and ^∗∗^*P* < 0.01, with respect to unpaired Student *t*-test. In B, statistical tests were performed at 10, 20, 40, and 60 s (dashed lines) and results indicated above the graphs.

To test the functional efficiency of integrins crosstalk on cell adhesion, we then performed local adhesiveness tests with beads coated with VCAM-1 and cells crawling on substrates coated either with VCAM-1 or with ICAM-1. Attachment (Figure 6A) and detachment (Figure 6B) experiments showed a higher adhesion on cell rear when cell basal side was engaged on VCAM-1 than on ICAM-1. These results support directly the existence of an inhibiting crosstalk of LFA-1 toward VLA-4 (Porter and Hogg, 1997; Grönholm et al., 2016) that is strong enough to induce a macroscopic change of adhesiveness phenotype (Figure 6C). We then performed equivalent local adhesiveness tests with ICAM-1-coated beads. Attachments (Figure 7A) and detachments (Figure 7B) showed higher adhesion in cell front for cell crawling on VCAM-1 than on ICAM-1. The same tendency is at the limit of significance on detachment experiments for cell rear. These results comfort the existence of an activating crosstalk of VLA-4 toward LFA-1 (Chan et al., 2000; May et al., 2000) (Figure 7C). In both cases, crosstalk signaling was active at long distances because the source, located at the adhesion zone of the cell on the substrate, and the target, located at the adhesion zones between beads and cells, are not co-localized at the molecular scale (Figures 6C, 7C).

### Crosstalks Require Engagement of Integrins With Ligands Anchored to a Solid Substrate

The strength of integrin crosstalk on cell surface adhesion suggests that the engagement of one integrin type to its ligand triggers strong inhibition or activation of the other integrin type. We then attempted to measure by cytometry the relative number of integrins in high affinity. However, we found no detectable change when ligands of the other integrin were added in solution, whereas a control with Mn^2+^ stimulated high affinity of LFA-1 and VLA-4, though to a lesser extent in the latter case (Supplementary Figure 2). These results with soluble ligands suggest that the anchoring of ligands to a solid substrate is necessary for the emitter integrins to send a crosstalk signal, and/or for the effector integrins to change their conformation. A similar requirement of ligand anchoring to a substrate was observed for the final activation of integrins stimulated by inside-out signals (Stewart et al., 1996; Constantin et al., 2000; Schürpf and Springer, 2011) and confirmed by our data in Supplementary Figure 2. Altogether, functional measurements of local adhesion reveal strong and long-range crosstalks between integrins VLA-4 and LFA-1, provided that both integrins are engaged with a solid substrate. The molecular mechanism of these crosstalks remains, however, unknown.

### Quantitative Optical Mapping of High-Affinity LFA-1 and VLA-4 Yields an Estimation of Bonds Number With a Substrate and Beads

**FIGURE 8:**
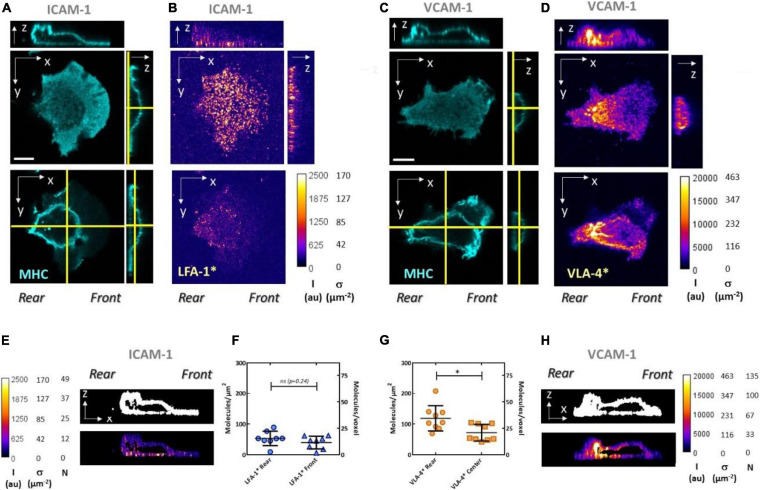
Quantitative confocal microscopy reveals high-affinity integrin distribution and density at the surface of crawling lymphocytes. Confocal imaging of cells crawling either on ICAM-1 **(A,B,E,F)** or VCAM-1 **(C,D,G,H)**. Immunostaining is used to reveal MHC proteins with Ab W6/32 **(A,C)**, integrins LFA-1 in high affinity with Ab M24 **(B)**, and β1 integrins (including VLA-4) in high affinity with Ab HUTS4 **(D)**. Top x–y images correspond to the basal plane, while bottom ones correspond to a mid-section of the cells. The heights where x–y images were taken are indicated by the yellow vertical lines in the corresponding y–z cross sections next to them. Section positions of the x–z and y–z images are indicated by yellow horizontal and vertical lines, respectively, in the bottom x–y images. Color scale bars are graduated for raw intensity, I (au), and surface density of high affinity integrins, σ (μm^−2^). White scale bar is 5 μm. Mask created from the x–z cross section of MHC (top) and immunofluorescence intensity within the mask (bottom) for high-affinity integrins LFA-1 **(E)** and VLA-4 **(H)**. Color scale bars are graduated for raw intensity, I(au), surface density of high-affinity integrins, s (μm^−2^), and number of molecules per voxel, N. Average density of high-affinity integrins measured at the basal plane, on cell front and rear for LFA-1 **(F)**, and on cell rear and center for VLA-4 **(G)**. Values are calculated based on the raw intensity on the basal plane minus the average background on the substrate. Ncells > 15. *P < 0.05 (two-tailed Student’s t-test).

In an attempt to directly observe and quantify the distributions of high-affinity integrins around cells and their modulation by signals issued by polarization, ligands, and crosstalk, we then performed quantitative confocal microscopy. To image the conformation of the cytoplasmic membrane, we stained the protein MHC, which diffuses at the cell membrane and is distributed on the whole-cell surface. Integrins LFA-1 and VLA-4 in high-affinity state, noted henceforth LFA-1^∗^ and VLA-4^∗^, were stained with antibodies M24 and HUTS4, respectively. M24 on live crawling cells induced a rapid staining of the adhesive basal cell zone (Supplementary Movie 3), followed by a strong impairment of uropod detachment and a global arrest of cells (Supplementary Movie 4). The known activating effect of M24 on integrin LFA-1 was thus strong enough to overcome the deactivation effect of polarization signaling and/or to hamper the internalization of LFA-1 in cell rear. These results exemplify some of the limitations of live immunostaining. They also suggest that imaging of cells that were first stained and then fixed necessarily corresponds to altered phenotypes. Therefore, all imaging here was performed on cells that were first fixed and then stained, to image cells in their normal phenotype. To quantify the intensity of confocal images, we then measured the voxel size by scanning 100-nm-diameter particles in the three dimensions, and we calibrated the intensity measured per voxel by imaging solutions of antibodies M24 and HUTS4 at known concentrations (Supplementary Figure 3 and section “Materials and Methods”). This method allowed us to estimate the absolute density of integrins in 3D at the surface of crawling human effector T lymphocytes (Figure 8).

The expected enrichment of LFA-1^∗^ in cell front was detectable on some images for cells adhering on ICAM-1 (Figures 8A,B) but not significant in systematic measurements of intensity at cell front and rear (Figure 8F). The polarization of VLA-4^∗^ toward cell rear was in turn detectable on all raw images for cells adhered on VCAM-1 (Figures 8C,D) and confirmed by measurements in cell rear and central zones (Figure 8G). A higher signal in the basal zone as compared to the dorsal zone, expected as a marker of activation by integrin ligands anchored to a solid substrate, was marked for LFA-1 (Figure 8E) but less pronounced for VLA-4 (Figure 8G). In terms of absolute number of integrins, the densities of 45 molecules.μm^−2^ found for LFA-1^∗^ are consistent with the average total LFA-1 density of 120 molecules.μm^−2^ measured by flow cytometry and suggest that a large fraction but not all LFA-1 molecules are activated in the basal plane. The density of VLA-4^∗^ was estimated to be 90 molecules.μm^−2^, which is higher than the average density of total VLA-4 measured by cytometry at 75 molecules.μm^−2^. The high concentration of high-affinity VLA-4 correlates with high concentration of MHC. Assuming that MHC is evenly distributed, excess of VLA-4 may therefore partly be explained by excess of membrane due to microvilli in cell rear. Accumulation of proteins in cell rear is widely observed in migrating cells and at times attributed to a ubiquitous drag of membrane material by the backward treadmilling of cortical actin (Rantal et al., 2011; Pouwels et al., 2013; Maiuri et al., 2015). Accumulation of VLA-4^∗^ may also result from a real excess of VLA-4 engaged with the substrate, which is consistent with a higher local adhesiveness mediated by VLA-4 on cell rear as compared to both LFA-1 in cell front (Figure 3) or LFA-1 associated with Mn^2+^ in cell rear (Figure 4). Furthermore, the fact that high-affinity LFA-1 imaging displayed less or no accumulation in cell rear and was more homogeneous in the whole basal plane suggests that recycling of integrins by endocytosis (Paul et al., 2015) in cell rear and frontward vesicular transport is particularly efficient for LFA-1. In the model of crawling/swimming propulsion by treadmilling/recycling mechanism (Bretscher, 1992; Aoun et al., 2020), a faster recycling of LFA-1 than VLA-4 is consistent with a higher crawling speed observed on substrates coated by ICAM-1 than by VCAM-1 (Hornung et al., 2020). Altogether, while integrin immunostaining experiments are delicate and highly dependent on the properties of antibodies and fixation/staining processes, they yield information at molecular level which is complementary to the mapping of local adhesion phenotypes at cell level using beads and flow experiments.

## Discussion

### A Novel Method to Map Adhesiveness on Live Cells

While the spatiotemporal regulation of integrin affinity and the associated local adhesiveness of the cell surface are crucial to mediating proper migration (Semmrich et al., 2005; Smith et al., 2005; Huttenlocher and Horwitz, 2011) and guidance (Carter, 1967; Valignat et al., 2013, 2014; Dominguez et al., 2015; King et al., 2016; Swaminathan et al., 2016; Hornung et al., 2020; Luo et al., 2020) functions, they remain partially unknown. Deciphering how integrins control migration phenotypes is difficult due to the complexity of a system that includes several types of integrins and several signaling pathways controlling integrin affinity in space and time. Another difficulty is that it is technical and consists in probing integrin affinity state or local surface adhesiveness on live cells without perturbing the migration/adhesion phenotype of interest. Our technique is in this context relevant because it directly assesses the adhesiveness of the surface on living cells with minimal perturbations. Other techniques such as atomic force microscopy (AFM) may provide similar information, even with higher spatial and temporal resolution. However, AFM is an expensive and sophisticated technique with limited throughput, whereas our method is inexpensive and low-tech and has a higher throughput.

### Functional Mapping of Local Adhesion Is Complementary to Molecular Analysis of Integrin State

From a fundamental point of view, method probing and mapping cell adhesiveness properties at cell scale by functional testing are instrumental to complementing methods examining the regulation of CAM at molecular scale, for instance, by immunostaining imaging, co-immunoprecipitation, specific inhibitors, gene silencing with RNAi, or cell-type-specific conditional knockouts. First, cell scale is directly relevant in understanding cell phenotypes of spreading, migration, or guidance, which are conditioned by the adhesion of cell leading and trailing edges to the environment. Mapping of cell surface adhesiveness gives therefore direct access to such information without having to decipher the underlying molecular mechanisms. Second, a molecular description of the state of adhesion molecules at cell membrane does not give access to the effective adhesion at cell scale. There is no comprehensive model yet to assess the effective adhesion resulting from a complex assembly of bonds. The finest molecular characterization of integrin types, densities, and state is insufficient to infer cell adhesion/migration properties. In turn, the combination of molecular and functional information may be instrumental to deciphering adhesion/migration properties from molecular to cell scale. We showed here that polarization and crosstalk signals on integrins can induce significant changes of adhesion at cell scale and that Sharpin affected deadhesion at cell scale, albeit moderately. Third, it is important to consider that integrin-mediated adhesion may require interactions with ligands attached to a solid substrate (Stewart et al., 1996; Constantin et al., 2000; Schürpf and Springer, 2011; Nordenfelt et al., 2016). A latent adhesiveness of cells may not be clearly revealed by immuno-imaging although integrins may actually be stimulated in an intermediate-affinity state. In contrast, our functional assay with solid beads directly assesses the capacity of the cell to interact with a substrate. As an example, immuno-imaging showed a lower activation of integrins at dorsal compared to basal surface with LFA-1, which suggested a lower adhesiveness of dorsal versus basal surface, but functional testing of local adhesion showed that the dorsal side of crawling cell was actually also adhesive. Fourth, molecular approaches have specific limitations. Antibodies against activated integrins often perturb their affinity, as exemplified here with M24, a marker of LFA-1 in high affinity. M24 instantly blocked the crawling of lymphocytes on substrates coated by LFA-1 ligands (Supplementary Movies 3, 4), which hampered M24 immuno-imaging of live crawling lymphocytes. In contrast, our method allowed testing cells in live conditions without perturbations. Fifth, molecular and functional measurements not only are complementary to link integrin properties and cell-scale adhesiveness but also shed light on properties that can only appear at large scales. For instance, molecular-scale data revealed polarization of integrin VLA-4 but not LFA-1 in the basal side, whereas cell-scale functional data by RICM (Pouwels et al., 2013; Valignat et al., 2014; Hornung et al., 2020) and bead experiments on crawling and swimming cells systematically revealed a polarization of VLA-4-mediated adhesion backward and of LFA-1-mediated adhesion frontward. This apparent discrepancy may be reconciled by considering different efficiencies of integrin recycling (via endocytosis and forward intracellular vesicular transport). A fast recycling of LFA-1 favors a homogeneous front–rear distribution, whereas a slower recycling favors an accumulation in cell rear. Altogether, functional mapping of adhesion yields complementary information to molecular studies, and the combination of these multiscale approaches is instrumental to shedding new light on the regulation network of integrins and on the adhesion/migration phenotype that they sustain.

### New Insight on the Global Model of the Integrin Regulation Network

**FIGURE 9:**
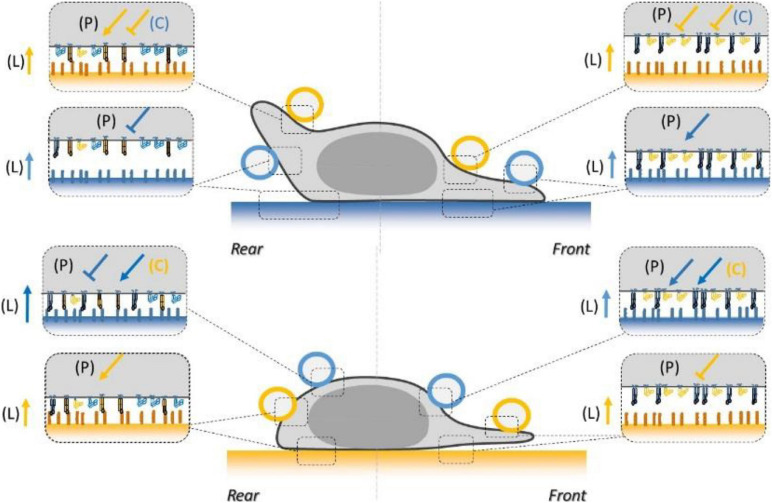
Schematics of the combined inside-out and outside-in signals regulating LFA-1 and VLA-4 integrin affinity at the basal and dorsal surface of a human effector T lymphocyte crawling on ICAM-1 substrate (blue) or VCAM-1 substrate (orange). Integrins are represented in orange for VLA-4 and blue for LFA-1, in their high- (extended, filled) and low- (bended, hollow) affinity states. Coatings on beads and substrates are colored in blue for ICAM-1 and orange for VCAM-1. Arrows and T-lines indicate, respectively, activation and inhibiting effects, and letters indicate their origin, i.e., the polarization machinery (P), ligand bonding (L), and crosstalk (C).

The functional testing of local adhesion allowed us to establish how integrins LFA-1 and VLA-4 globally mediate the adhesiveness of the surface of crawling lymphocytes (Figure 9). Local adhesiveness varies for each integrin with the location on the cell due to a combination of polarization signaling (P), an apparent outside-in effect by ligands anchored to a substrate (L), and multiple crosstalk signaling (C). The local enhancement or decrease of adhesiveness is represented in Figure 9 by an arrow and a T line, respectively, which is reminiscent of the representation used in functional protein networks. However, if a relatively higher or lower integrin-mediated adhesiveness suggests a corresponding higher or lower integrin affinity, it does not necessarily imply the existence of a, respectively, activating (arrow) and inhibiting (T-line) signal on integrins. A first scenario with a global resting state of integrins in low affinity and a single activating signal modulated throughout the cell is, for instance, sufficient to tune integrins into higher or lower state according to a lower or higher intensity of the inhibiting signal. A second opposite scenario with a high-affinity resting state and a single activating signal can yield a similar output. A third and more complex scenario with multiple inhibiting and activating signals is also plausible. The canonical models of integrin activation have long favored the first scenario of inactive integrin conformation adopted by the receptor in the absence of activating proteins, and inside-out activation signals to modulate adhesion throughout the cell (Shattil et al., 2010). Transition of integrins from the inactive state to the active state and linkage of integrins to the cytoskeleton indeed imply signaling by Rap1 GTPase and its effector Rap1-interacting adaptor molecule (RIAM), which induces direct binding of proteins such as Talin, Kindlin, and FAK to β-chain integrin tails (Shimonaka et al., 2003; Lafuente et al., 2004; Calderwood et al., 2013). This dogma of constitutively low-affinity resting state for integrins was then challenged by increasing evidences that integrin-inactivating signals were also crucial for appropriate cell functions (Semmrich et al., 2005) *in vivo* and *in vitro*. These signals were proposed to imply various proteins such as Myosin, Sharpin, Icap-1, Filamin 1, and Shank (Rantal et al., 2011; Bouvard et al., 2013; Liu et al., 2015; Lilja et al., 2017). The idea that inactive LFA-1 was the passive default form was then directly challenged in lymphocytes by the finding that active interaction of endogenous Sharpin with α_*L*_-tail of LFA-1 integrins was required to maintain a non-activated state (Rantal et al., 2011). Our results support a role of Sharpin in deactivation; however, deactivation of LFA-1 and uropod detachment was still possible in human effector T lymphocytes deficient for Sharpin. This difference may be explained by a difference between human-effector versus mouse-naïve lymphocytes, or by insufficient Sharpin deficiency in our experiments. Altogether, these results support the inexistence of a resting or reference state for integrins, and the validity of the third scenario in which the regulation of integrin–ligand interactions results from a finely tuned balance between activating and inhibiting signals. Several observations are in line with these conclusions. Sharpin was found effective throughout the cell, not just in cell rear, so that cell front is hosting both activating and inhibiting signaling processes. It is then tempting to hypothesize that the whole cell is hosting the same set of signaling cascades, each being differently modulated in space and time. This is consistent with the observations of an increasing concentration gradient of Sharpin toward cell rear (Pouwels et al., 2013) and of activating Rap-1 toward cell front (Yi et al., 2012). The resulting adhesiveness of the surface of a cell would in the end be set by a local equilibrium between all biochemical signaling reactions. Our data also support a “solid substrate effect” on the activation of integrins [called (L) in this work]. This effect was evidenced by immune-imaging showing more activated LFA-1 in basal versus apical side. The mechanism underlying this solid-substrate effect is unknown but seems independent of actomyosin contractility since bead deadhesion was not affected by treatment with blebbistatin or Y27632.

### Integrin Regulation Network Can Explain the Emergence of Sophisticated Adhesion/Migration Phenotypes

The comprehensive description of lymphocyte local adhesiveness summarized in Figure 9 is crucial to explaining specific and sophisticated phenotypes of adhesion and migration of lymphocytes previously reported in the literature. First, the slower migration of crawling cells on VCAM-1 as compared to ICAM-1, as well as the attachment of the uropod on VCAM-1 and its detachment on ICAM-1, is consistent with a polarization of cell adhesiveness toward cell rear on VCAM-1 and cell front on ICAM-1. Second, the guidance mechanism of lymphocytes with or against a flow versus the composition of the substrates in VCAM-1 and ICAM-1 was qualitatively explained by a bistable mechanism relying on an exquisite regulation of LFA-1 and VLA-4 affinity along the cell front–rear axis (Hornung et al., 2020). In the bistable system, cells had the choice between two states; in the first state, cells had with their front attached and their rear detached, which yielded upstream phenotype, and conversely in the second state, they had their front detached and their rear attached, which yielded a downstream phenotype. The two states can emerge from the opposite polarizations of LFA-1 and VLA-4 affinity coupled to further modulation by crosstalk between LFA-1 and VLA-4. Molecular findings on integrin polarization (Laudanna et al., 1996; Semmrich et al., 2005; Smith et al., 2005, 2007; Ghandour et al., 2007; Morin et al., 2008; Shulman et al., 2009; Hogg et al., 2011; Rantal et al., 2011; Pouwels et al., 2013; Valignat et al., 2013, 2014; Grönholm et al., 2016; Hornung et al., 2020) and crosstalk (Porter and Hogg, 1997; Chan et al., 2000; May et al., 2000; Grönholm et al., 2016) were already consistent with the bistable model. Our functional measurements now directly confirm that adhesiveness is indeed modulated at cell scale in accord with the bistable model. These data support further a controversial proposition that guiding of lymphocytes under flow does not require mechanotransduction by integrins and more generally that a complex regulation network of integrins can trigger the emergence of sophisticated guidance mechanisms without mechanotransduction by integrins.

### Toward a Quantitative Understanding of Integrin-Mediated Adhesion and Associated Phenotypes

Our approach can shed light on the biochemical efficiency of the integrin regulation process at cell scale, but it is also directly relevant to decipher the mechanical efficiency in terms of adhesion strength of a complex assembly of multiple integrin bonds. 3D quantitative immunostaining gives access to the densities of high-affinity integrins and laminar flow with beads to local adhesion, which provides the main ingredients to test physical models of adhesion in a multiple-bond system. In the future, systematic variation of the density of ligands on beads and on substrates may provide further information on the relation between adhesion strength and bond number, as well as on the quantitative effects of ligand activation and crosstalk. Altogether, we envision that pursuing systematic studies combining genome edition, quantitative immunostaining, and functional mapping of local adhesiveness will be instrumental to further deciphering the molecular mechanisms of integrin regulation and their output at cell scale on adhesion, migration, and guidance phenotypes.

## Materials and Methods

### Cells and Reagents

Whole blood from healthy adult donors was obtained from the Etablissement Francais du Sang. Peripheral blood mononuclear cells (PBMCs) were recovered from the interface of a Ficoll gradient (Eurobio, Evry, France). T cells were isolated with Pan T cell isolation Kit (Miltenyi Biotec, Bergisch Gladbach, Germany) and then activated for 2 days with T Cell TransAct^TM^ (Miltenyi Biotec, Bergisch Gladbach, Germany), a polymeric nanomatrix conjugated to humanized CD3 and CD28 agonists. Cells were subsequently cultivated in Roswell Park Memorial Institute Medium (RPMI; Gibco by Thermo Fischer Scientific, Waltham, MA, United States) 1640 supplemented with 25 mM GlutaMax (Gibco by Thermo Fischer Scientific, Waltham, MA, United States), 10% fetal calf serum (FCS; Gibco by Thermo Fischer Scientific, Waltham, MA, United States) at 37°C, and 5% CO_2_ in the presence of IL-2 (50 ng/mL; Miltenyi Biotec, Bergisch Gladbach, Germany) and used 7 days after activation. At the time of use, the cells were >99% positive for pan-T lymphocyte marker CD3 and assessed for activation and proliferation with CD25, CD45RO, CD45RA, and CD69 makers as judged by flow cytometry. ROCK inhibitor Y-27632 dihydrochloride was obtained from Sigma–Aldrich (St. Louis, MO, United States) and Myosin II inhibitor Blebbistatin from Fisher Bioblock Scientific (Illkirch, France).

### CRISPR-Cas9-Based Genome Editing of Human Primary T Cells

Sharpin-deficient human primary T cells were established using the following sgRNA-specifying oligonucleotide sequences to delete exon 1 (transcript ID: ENST00000398712.6): 5′-ACCGGAGATGGCGCCGCCAG-3′ and 5′-GGACCCGGCCGGACCGGAGATGG-3′. The Cas9 ribonucleoprotein (RNP) complex contains duplex of these crRNA with a transactivating RNA (tracrRNA). This duplex is then associated with Cas9 enzyme to form the RNP complex (RNA and enzyme supplied by IDT). The PCR product containing a full-length GFP sequence with homologous sequence of the human *Sharpin* locus was used to be inserted in the genome by homologous recombination. Cas9 RNPs and GFP PCR product were co-transfected into primary T cells using a Neon transfection kit and Device (Invitrogen). A control was produced with RNPs lacking of crRNA (transfected cell control). This GFP expression was used both as a control for Sharpin knock-out expression and as a marker for sorting Sharpin-KO cells. The average expression of cells before sorting was downregulated to 30% (Supplementary Figure 1).

### Flow Channel Preparation

Channels Ibidi μ-Slide VI^0.1^ (Ibidi GMBH, Martinsried, Germany) were coated at 4°C with 50 μL of a 10-μg/mL human ICAM-1-Fc or VCAM-1-Fc (R&D Systems, Minneapolis, MN, United States) in phosphate-buffered saline (PBS) (Gibco), rinsed three times with PBS, then blocked with 75 μL of a 2% bovine serum albumin (BSA, Sigma–Aldrich) solution in PBS (Life Technologies) for 25 min, and rinsed again three times with PBS, and finally filled with RPMI before injection of cells.

### Fluorescent Quantification of Adhesion Molecules on Substrates

PE-labeled Anti-Human CD54 (ICAM-1) and Anti-Human CD106 (VCAM-1) antibodies (eBioScience by Thermo Fischer Scientific, Waltham, MA, United States) were used for adhesion molecule quantification. First, we set up a bulk calibration curve by measuring the fluorescence intensity of antibody solutions inside thin channels of 48 μm in height at concentrations of 0, 1.5, 3, 5, and 7 μg/mL. Channels were pretreated with 1% Pluronic F127^©^ (Sigma–Aldrich, St. Louis, MO, United States) to limit the adsorption of antibodies on the channel surface. In the end, channels were rinsed extensively with PBS. Residual fluorescent intensity due to adsorbed antibodies was measured and then subtracted from the previous measurements. A previous study (Hornung et al., 2020) showed a linear relation between the fluorescent intensity and the bulk concentration. We assume that the signal is given by the total number of molecules in the thin channel, and then the volume concentration can be converted to a surface concentration for a channel of 48 μm in height. Then, for each sample used for cell adhesion and migration assay, the patterned surfaces coated with ICAM-1 or VCAM-1 were first rinsed extensively with cold PBS solution. Then, the sample was stained with a corresponding antibody at 10 μg/mL and incubated overnight at 4°C. Images were taken the next day with the Zeiss Z1 microscope setup. The fluorescent intensity was analyzed with ImageJ software (U.S. National Institutes of Health, Bethesda, MD, United States) at five different positions. The average intensity was converted into surface density of the adhesion molecules according to the calibration data.

### Bead Preparation

A 1-mL Eppendorf tube was pretreated for 15 min with 500 μL of a 4% BSA solution in PBS and then rinsed twice with 1 mL PBS to avoid adhesion of beads on the walls. In this antifouling-treated Eppendorf tube, a 5-μL solution of microbeads at 10 mg/mL (Dynabeads^TM^ M-280 Streptavidin, Invitrogen, 11205D) was washed three times using a magnet with a 0.1% BSA solution in PBS. Beads were then incubated in 500 μL of 0.1% BSA solution in PBS with 2 μL of a 1-mg/mL protein A-Biotin solution in PBS for 1 h at room temperature, washed three times with a 0.1% BSA solution in PBS, then incubated in 500 μL of 0.1% BSA solution in PBS with 3.8 μL of a 500-μg/mL solution of ICAM-Fc or VCAM-Fc for 2 h with continuous steering and, at room temperature, washed again three times, and finally stored as a stock solution in 500 μL of a 1% BSA solution in PBS. To modulate the density of Fc-ICAM or Fc-VCAM anchored on beads, the step of bead coating by Fc-CAM was performed by incubation in mixtures of Fc-CAM and human Immunoglobulins IgG (Tegeline, LFB Biomedicaments) at volume ratio 1/5. For flow experiment, 150 μL of the bead stock solution was mixed with 200 μL of RPMI. Quantification of ICAM-1 and VCAM-1 by quantitative cytometry using a secondary antibody and calibration beads (CellQuant calibrator kit, ref 7208, Biocytex) yielded an average number of 60,000 CAM molecules per bead.

### Quantitative Immunostaining Confocal Microscopy

Cells were incubated for at least 10 min on ICAM-1- or VCAM-1-coated microchannels and fixed by flowing 4% paraformaldehyde. Samples were rinsed after a 10-min incubation and stained with 1/50 dilution of either anti-CD11a/CD18-activated clone M24 (BioLegend) or anti-Integrin β1-activated clone HUTS4 (Merck). A 1/50 dilution of anti-human HLA-A,B,C clone W6/32 (BioLegend) was used in both cases as a counter-stain. After 30 min staining at room temperature, VCAM-1 samples were rinsed and mounted with Mowiol; ICAM-1 samples underwent a second fixation for 10 min with 1% PFA, to avoid antibody detachment, prior to mounting. For antibody calibration, a series of microchannels were prepared by blocking the surface with Pluronics F-127, to avoid surface adsorption, and a serial dilution of each antibody was imaged with the same settings as for the cells. Imaging was performed on a Zeiss LSM 880 Fast AiryScan microscope. The size of the voxel in our imaging conditions was determined by imaging fluorescent submicrometric beads (Molecular Probes^TM^ TetraSpeck^TM^, 0.1 μm) in the three directions of space (Supplementary Figure 3A). The voxel had an ellipsoid shape with main axis a = 589 nm and b = 539 nm in the plane perpendicular to the optical axis and c = 1519 nm along the optical axis. The intensity in a voxel was then calibrated by imaging solutions of the antibody of interest at different known concentrations (Supplementary Figure 3B). The absolute number of molecules in a voxel N of intensity I was then determined according to the equation:

(1) $$N=\frac{4}{3}\pi abc\frac{N_{a}I}{\alpha M}10^{-18}$$

where *Na* is the Avogadro number, *M* is the molar mass of the antibody with unit g/Mol, and α is the slope of the calibration curve *I* versus the concentration antibody of interest with unit mL/μg. The density of molecules per μm^2^ in the plane perpendicular to the optical axis, σ, is determined as:

(2) $$\sigma =\frac{4}{3}\pi c\frac{N_{a}I}{\alpha M}10^{-18}$$

The density of molecules σ was calculated here in the basal side of cells.

### Local Adhesiveness Measurement by Flow Experiments

Flow chambers (IBIDI, VI 0.1) coated with CAM molecules were filled with 50 μL of 7 × 10^6^ cells/mL suspension and connected to a 5-mL glass air-tight syringe actuated by a homemade system made of a syringe pump and a camera piloted by an Arduino Uno Rev3 (Arduino, Italy)-based controller allowing acquisition sequences at different shear rates. The temperature of the whole setup, including chambers, tubing, syringes, and microscope, was regulated at 37°C. Cells were settling in the chambers without flow for 15 min, then beads were injected, and flow sequences were started either for detachment experiments (shear stress sequences of 0.02 dyn/cm^2^ for 3 min and 4 dyn/cm^2^ for 1 min) or for attachment experiments (continuous shear stress of 0.04 dyn/cm^2^). Video acquisition was made at 25 frames/s on a Zeiss inverted microscope (Observer Z1, Zeiss) in bright-field mode with a ×10 magnification objective (UPlanApo 10×/0.40, Olympus) and a UI3360-M-GL camera (IDS, Germany).

### Data Analysis of Local Adhesiveness Data by Flow Experiments

Microsphere attachment and detachment data were gathered in a semi-automated way. First, microsphere trajectories were retrieved using a program written in Java (Oracle, United States) for ImageJ (National Institutes for Health, United States) that formed trajectories from microsphere positions using a proximity criterion from one movie frame to the next one. A second program written in Java for ImageJ detected microsphere arrests (using a velocity threshold criterion) and microsphere lateral deviation from shear flow-induced straight paths (using a *Y*-axis motion threshold criterion).

For attachment detection, experiments were done at a constant shear stress of 0.04 dyn/cm^2^. Two kinds of events were collected from microsphere trajectories: either arrests (that may have been triggered by interaction of microspheres with cells) or lateral motions usually induced by microsphere encounter with a cell. A third program written in Java for ImageJ used these data and the experimental movie to present a graphic user interface that showed for each of those events a close-up of the microsphere from the experimental movie, with also a view of the whole movie set at the frame when the event occurred, with the area surrounding the microsphere highlighted. The operator then immediately chose whether the event was a false positive (caused by non-specific adhesion events of microsphere to the chamber surface) and discarded it, or a relevant microsphere–cell interaction. The operator could then replay the movie around the frame of the relevant event, to assess the position of microsphere–cell contact relatively to the cell. Contact could be on the lamellipod, or on the uropod, or on the central cellular body. Location of cell contact was decided by the operator based on the cell morphology and, mostly, on the direction of cell migration, observed by replaying the movie. Dubious cases (usually non-motile cells and round cells) were discarded. Finally, the operator controlled whether the contact was followed by microsphere–cell adhesion or not. Data were collected as a table showing identity of the trajectory, position of the contact relatively to the cell, and a Boolean indicating adhesion or non-adhesion. Adhesion frequency was defined as the ratio, on a given cell location (lamellipod, central cellular body, and uropod), of contacts followed by adhesion over total number of contacts.

For detachment measurement, experiments were performed using a low shear stress period of 0.02 dyn/cm^2^ allowing microsphere–cell contact (and eventually adhesion), followed by a high shear stress period of 4 dyn/cm^2^. Microsphere trajectories were retrieved during the high shear stress period only. Relevant events were microsphere–cell adhesion events already set when the high shear stress started; lateral motion and later arrests were not considered. A second mode of the same third program presented the same graphic user interface that allowed the operator to, first, decide whether the event was a false positive and discard it, and to assess the position of microsphere–cell contact relatively to the cell, with criterions identical to attachment experiments. Finally, the operator then controlled whether the microsphere–cell adhesion broke during the high shear stress period or survived it, and checked and eventually corrected automated measurement of the dates of beginning and end of microsphere–cell adhesion. Data were collected as a table showing the identity of the trajectory, position of the contact relatively to the cell, and dates of beginning and end of microsphere–cell adhesion giving duration of each microsphere–cell adhesion event. Detachment for a given condition was quantified by building microsphere–cell adhesion event survival curves that displayed the proportion of surviving adhesion events versus their duration.

## Data Availability Statement

The original contributions presented in the study are included in the article/Supplementary Material. Further inquiries can be directed to the corresponding author/s.

## Author Contributions

NG-S, PH, DT, OT, and PR performed experiments for quantitative assays with beads; M-PV, PR, and DT for cell attachment; and NG-S and SB for quantitative confocal imaging. MB-P managed the control of cell culture, performed quantitative characterizations by cytometry, and prepared/analyzed Sharpin deficient primary human T cells. BM conceived the method to perform gene edition in human primary T cells using CRISPR-Cas9. PN and MB-P developed it experimentally. M-PV participated in experiments, analysis, and project design. PR conceived and built the automatized laminar flow setup for bead detachment and the analysis code. PR and OT designed the project and supervised the experiments and analysis. OT wrote the manuscript. All authors contributed to the article and approved the submitted version.

## Conflict of Interest

The authors declare that the research was conducted in the absence of any commercial or financial relationships that could be construed as a potential conflict of interest.

## Supplementary Material

Supplementary Movie 1 Attachment and detachment of beads on a cell crawling on ICMA-1-coated substrate. ICAM-1-coated beads attach on cell during the low flow sequence, are advected backward by treadmilling, and eventually detach during high flow sequence.

Supplementary Movie 2 Attachment and detachment of beads on a non-adherent swimming cells in the absence of flow; first with ICAM-1-coated beads and then with VCAM-1-coated beads. Beads are advected to cells trailing edge, where they eventually detach spontaneously for ICAM-1-coated beads and conversely accumulate with VCAM-1-coated bead.

Supplementary Movie 3 Lymphocytes crawling on ICAM-1-coated substrates with antibody M24 at 10 μg/mL before time 8 min and without M24 after 8 min image in Bright field (left) and fluorescence mode (Right). Cells are motile and have a detached uropod after rinsing, whereas cells with M24 are arrested with an adherent uropod enriched in high affinity LFA-1 integrins.

Supplementary Movie 4 Lymphocytes crawling on ICAM-1-coated substrates without (top) and with (bottom) antibody M24 at 10 μg/mL, and observed in Bright field (left) and reflection interference contrast microscopy (RICM) mode (right). Control cells are motile and have a detached uropod, whereas cells with M24 are arrested with an adherent uropod.
