# Supplementary material for: Functional Mapping of Adhesiveness on Live Cells Reveals How Guidance Phenotypes Can Emerge From Complex Spatiotemporal Integrin Regulation
Source: Front Bioeng Biotechnol. 2021 Apr 7;9:625366. doi: 10.3389/fbioe.2021.625366 (PMC8058417; doi:10.3389/fbioe.2021.625366)
Supplement: Supplementary file 5 [file Data_Sheet_1.docx]

# SUPPLEMENTARY MATERIAL

#### Supplementary videos

*Movie 1: Attachment and detachment of beads on a cell crawling on ICMA-1 coated substrate. ICAM-1 coated beads attach on cell during the low flow sequence, are advected backwards by treadmilling and eventually detach during high flow sequence.*

Movie 2: Attachment and detachment of beads on a non-adherent swimming cells in absence of flow; first with ICAM-1 coated beads then with VCAM-1 coated beads. Beads are advected to cells trailing edge, where they eventually detach spontaneously for ICAM-1 coated beads and conversely accumulate with VCAM-1 coated bead.

Movie 3: Lymphocytes crawling on ICAM-1 coated substrates with antibody M24 at 10 µg/mL before time 8 min and without M24 after 8 min image in Bright field (left) and fluorescence mode (Right). Cell are motile and have a detached uropod after rinsing whereas cells with M24 are arrested with an adherent uropod enriched in high affinity LFA-1 integrins.

Movie 4: Lymphocytes crawling on ICAM-1 coated substrates without (top) and with (bottom) antibody M24 at 10 µg/mL, and observed in Bright field (left) and reflection interference contrast microscopy (RICM) mode (Right). Control cell are motile and have a detached uropod, whereas cells with M24 are arrested with an adherent uropod.

#### Supplementary Figures


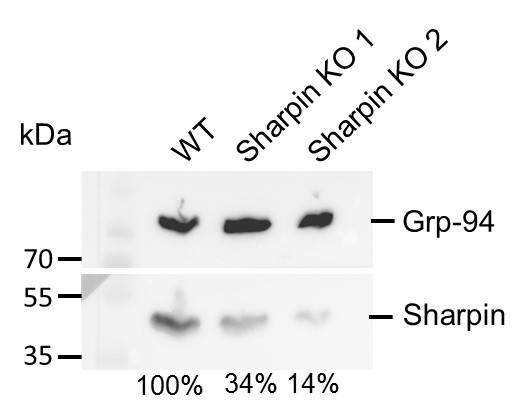


Supporting Fig. S 1: Generation of Sharpin deficient Human effector T cells. Immunoblot analysis of equal amounts of total lysates of primary human effector T lymphocytes 1- Control cells. 2 and 3- Cells silenced by Crispr-Cas9 with two different primers (see material and method) before sorting by GFP expression. Percentage correspond to relative expression of Sharpin.


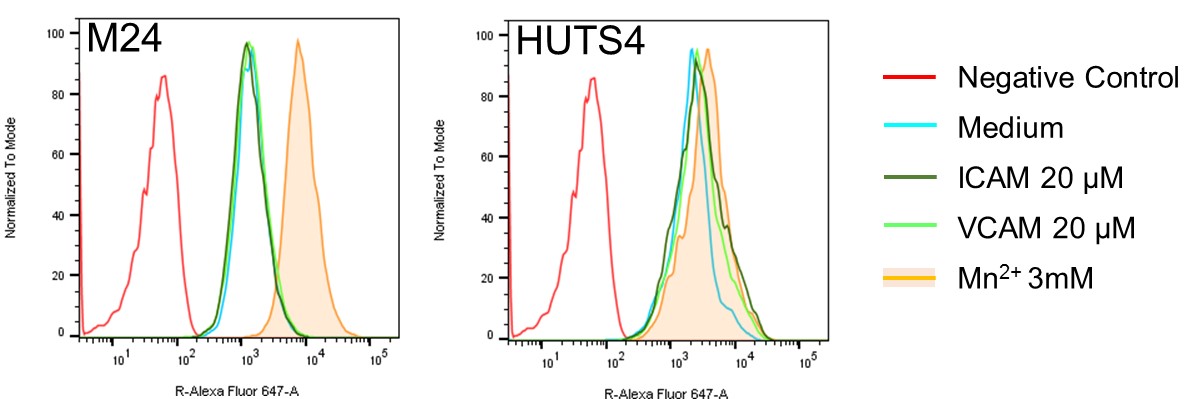


Supporting Fig. S 2: Absence of detectable crosstalk with soluble integrin ligands. Cytometry histograms of effector T cells in control medium, with soluble ICAM or VCAM at 20 µM and with Mn^2+^ at 3mM with antibody M24 against high affinity LFA-1 (left) and antibody HUTS4 against high affinity VLA-4 (right).


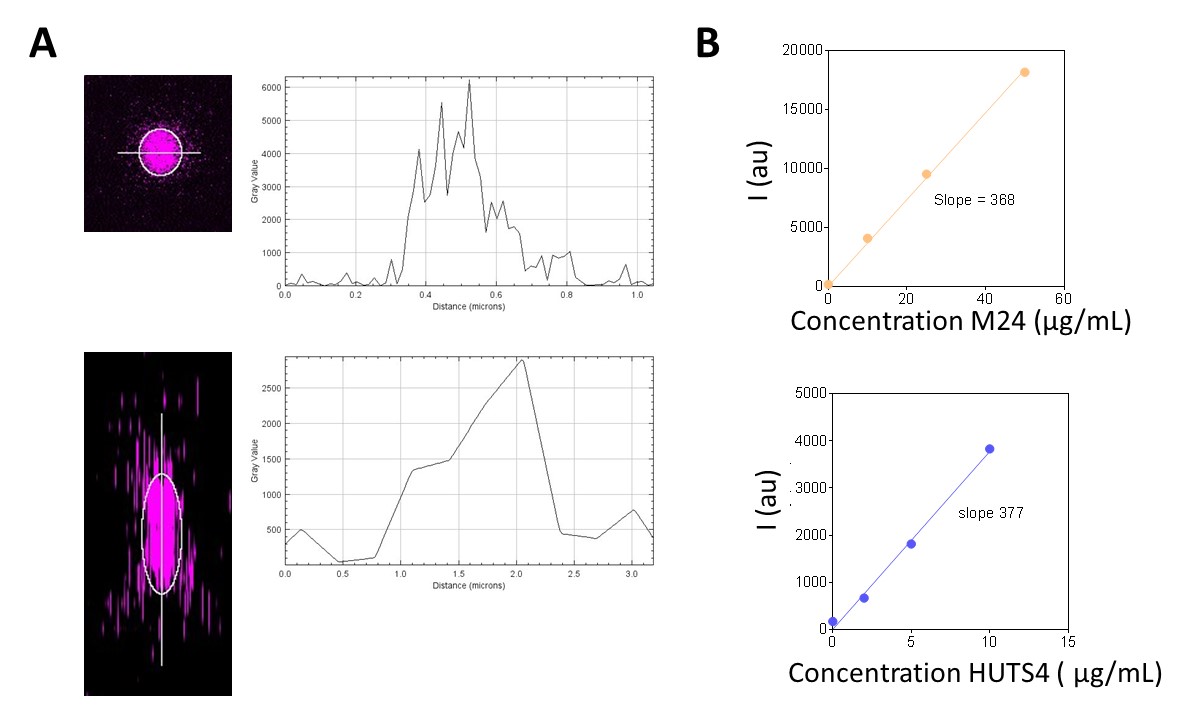


Supporting Fig. S 3: **Calibration of confocal microscopy images** A) Top (xy) and side (xz) views of a nanobead used to calculate confocal excitation volume. Linear ROI's are used to plot signal intensity along x and z axis, circular ROI's are used to calculate the 3D ellipsoid volume, by using its characteristic major and minor axis, reaching a 2 um3 excitation volume. B) Calibration curves for M24 and HUTS4 antibodies. Serial dilutions of each antibody were imaged with the same conditions as for cells, and the fluorescent intensity was plotted as a function of antibody concentration. The slope (alpha) was then used in equations 1 and 2 to convert fluorescent signal into number of staining molecules.
